# Short-Term Clinical Response and Changes in the Fecal Microbiota and Metabolite Levels in Patients with Crohn’s Disease After Stem Cell Infusions

**DOI:** 10.1093/stcltm/szad036

**Published:** 2023-07-03

**Authors:** Fan Yang, Xiaofang Zheng, Weicheng Liang, Beibei Ni, Jianxi Lu, Qiuli Liu, Ruixuan Xu, Yizhan He, Mary Miu Yee Waye, Qi Zhang, Yufeng Chen, Xiaoguang Zou, Wenjie Chen

**Affiliations:** Biotherapy Centre, The Third Affiliated Hospital, Sun Yat-sen University, Guangzhou, People’s Republic of China; Department of Infectious Diseases, The First People’s Hospital of Kashi, The Kashi Affiliated Hospital, Sun Yat-Sen University, Kashi, People’s Republic of China; Postdoctoral Research Station, Xinjiang Medical University, Ürümqi, People’s Republic of China; Biotherapy Centre, The Third Affiliated Hospital, Sun Yat-sen University, Guangzhou, People’s Republic of China; Cell-Gene Therapy Translational Medicine Research Centre, The Third Affiliated Hospital, Sun Yat-sen University, Guangzhou, People’s Republic of China; Cell-Gene Therapy Translational Medicine Research Centre, The Third Affiliated Hospital, Sun Yat-sen University, Guangzhou, People’s Republic of China; Biotherapy Centre, The Third Affiliated Hospital, Sun Yat-sen University, Guangzhou, People’s Republic of China; Cell-Gene Therapy Translational Medicine Research Centre, The Third Affiliated Hospital, Sun Yat-sen University, Guangzhou, People’s Republic of China; Biotherapy Centre, The Third Affiliated Hospital, Sun Yat-sen University, Guangzhou, People’s Republic of China; Biotherapy Centre, The Third Affiliated Hospital, Sun Yat-sen University, Guangzhou, People’s Republic of China; Biotherapy Centre, The Third Affiliated Hospital, Sun Yat-sen University, Guangzhou, People’s Republic of China; The Nethersole School of Nursing, The Chinese University of Hong Kong, Shatin, Hong Kong SAR, People’s Republic of China; Biotherapy Centre, The Third Affiliated Hospital, Sun Yat-sen University, Guangzhou, People’s Republic of China; Department of Infectious Diseases, The First People’s Hospital of Kashi, The Kashi Affiliated Hospital, Sun Yat-Sen University, Kashi, People’s Republic of China; Cell-Gene Therapy Translational Medicine Research Centre, The Third Affiliated Hospital, Sun Yat-sen University, Guangzhou, People’s Republic of China; Department of Colorectal Surgery & Department of General Surgery & Guangdong Provincial Key Laboratory of Colorectal and Pelvic Floor Diseases, The Sixth Affiliated Hospital, Sun Yat-sen University, Guangzhou, People’s Republic of China; Department of Infectious Diseases, The First People’s Hospital of Kashi, The Kashi Affiliated Hospital, Sun Yat-Sen University, Kashi, People’s Republic of China; Biotherapy Centre, The Third Affiliated Hospital, Sun Yat-sen University, Guangzhou, People’s Republic of China; Department of Infectious Diseases, The First People’s Hospital of Kashi, The Kashi Affiliated Hospital, Sun Yat-Sen University, Kashi, People’s Republic of China; Cell-Gene Therapy Translational Medicine Research Centre, The Third Affiliated Hospital, Sun Yat-sen University, Guangzhou, People’s Republic of China; NMPA Key Laboratory for Quality Research and Evaluation of Cell Products, Guangzhou, People’s Republic of China

**Keywords:** umbilical cord (UC), mesenchymal stem cells (MSCs), cellular therapy, autoimmune disease, clinical translation

## Abstract

Recent studies have shown a close relationship between the gut microbiota and Crohn’s disease (CD). This study aimed to determine whether mesenchymal stem cell (MSC) treatment alters the gut microbiota and fecal metabolite pathways and to establish the relationship between the gut microbiota and fecal metabolites. Patients with refractory CD were enrolled and received 8 intravenous infusions of MSCs at a dose of 1.0 × 10^6^ cells/kg. The MSC efficacy and safety were evaluated. Fecal samples were collected, and their microbiomes were analyzed by 16S rDNA sequencing. The fecal metabolites at baseline and after 4 and 8 MSC infusions were identified by liquid chromatography-mass spectrometry (LC--MS). A bioinformatics analysis was conducted using the sequencing data. No serious adverse effects were observed. The clinical symptoms and signs of patients with CD were substantially relieved after 8 MSC infusions, as revealed by changes in weight, the CD activity index (CDAI) score, C-reactive protein (CRP) level, and erythrocyte sedimentation rate (ESR). Endoscopic improvement was observed in 2 patients. A comparison of the gut microbiome after 8 MSC treatments with that at baseline showed that the genus *Cetobacterium* was significantly enriched. Linoleic acid was depleted after 8 MSC treatments. A possible link between the altered *Cetobacterium* abundance and linoleic acid metabolite levels was observed in patients with CD who received MSCs. This study enabled an understanding of both the gut microbiota response and bacterial metabolites to obtain more information about host-gut microbiota metabolic interactions in the short-term response to MSC treatment.

## Graphical abstract

**Graphical Abstract:**
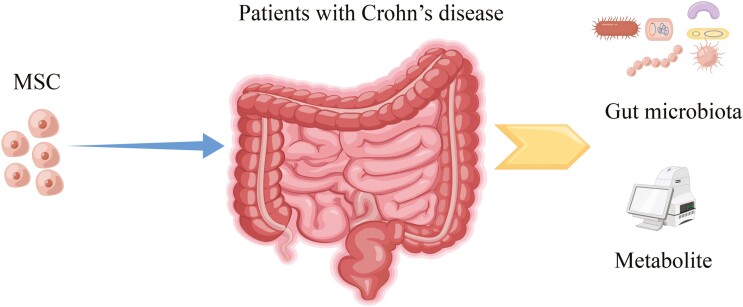

**Lessons Learned**

- MSC infusions are safe and feasible for patients with Crohn’s disease (CD).
- The clinical symptoms and signs of CD patients significantly improved after 8 MSC infusions.
- A possible link between the altered *Cetobacterium* abundance and linoleic acid metabolite levels was observed in patients with CD who received MSCs.

**Significance Statement**

Our study provides the first documentation of the changes in the gut microbiota of patients with relapsing refractory CD after MSC treatment. This study enabled an understanding of both the gut microbiota response and bacterial metabolites to enhance information about host--gut microbiota metabolic interactions in the short-term response to MSC treatment. These data suggest that the gut microbiota could be used as a promising diagnostic biomarker to evaluate the prognostic efficacy and therapeutic targets of MSCs.

## Introduction

Inflammatory bowel diseases (IBDs), consisting of Crohn’s disease (CD) and ulcerative colitis (UC), are chronic immune-mediated gut inflammatory diseases with a relapsing-remitting course.^1^ Patients with chronic relapsing CD have persistent transmural inflammation along the gastrointestinal tract, which requires chronic immunosuppression.^2,3^ Genetics is likely to play a role in CD. More than 30 distinct genomic loci are involved in the genetic susceptibility to CD.^4^ An infection or environmental factor may trigger or aggravate the disease.^5^ CD is treated with corticosteroids (CSs), immunomodulatory agents, and “biologic therapies,” including anti-TNF-α antibodies, vedolizumab, and ustekinumab.^6-9^ The standard anti-inflammatory regimen, however, does not halt disease progression.^10^ The CD remission rates must be improved, and recurrences must be reduced.

Mesenchymal stem cells (MSCs), which have anti-nflammatory, immunomodulatory and antifibrotic properties, potentially ameliorate both the inflamed and fibrotic components of CD.^11-13^ In theory, MSCs inhibit intestinal inflammation, promote long-term mucosal healing, and improve patients’ quality of life, which suggests that they are an excellent treatment option for CD.^14,15^ Several studies have evaluated the safety and effectiveness of CD stem cell therapy, but the results remain ­controversial.^16-19^ We aimed to assess the short-term safety and efficacy of MSC infusions in patients with relapsing refractory CD.

CD is presumed to develop as a result of environmental, microbial, and immune-mediated factors in genetically vulnerable hosts.^20^ Microbiomes may play an important role in CD development, progression, and treatment.^21-25^ A decrease in the abundance of *Faecalibacterium prausnitzii* in the ileum may contribute to a higher rate of postoperative recurrence of ileal CD and endoscopic recurrence at 6 months.^21,26^ IBD pathogenesis may be associated with the gut microbiota, but the precise role of dysbiosis is unclear. We compared the microbial communities in patients with CD before and after a course of MSC therapy to determine how MSCs affected the gut microbiota. This study provides a comprehensive picture of the microbiota in patients with CD and new insights into robust biomarkers that will aid in future studies of dysbiosis.

## Materials and Methods

### Study Design and Population

We conducted a prospective observational study between April 2018 and December 2019. All participants provided informed consent, and the study was approved by the Stem Cell Research Ethics Committee of The Third Affiliated Hospital of Sun Yat-sen University, China (approval No. [2017]-37), on September 27, 2017. Patients with relapsing refractory CD who initiated MSC therapy were consecutively recruited at the outpatient clinic of the biotherapy center at the Third Affiliated Hospital of Sun Yat-sen University. The inclusion criteria were as follows: (1) adult men and women aged between 18 and 70 years; (2) individuals diagnosed with CD based on clinical symptoms and the results of imaging studies, as well as endoscopic and pathological examinations, patients in the mild-to-moderate active phase (150 ≤ CD activity index (CDAI) < 450) and with evidence of active inflammation, and patients with relapsing refractory CD who failed to respond to corticosteroids, azathioprine, methotrexate, and antitumor necrosis factor (TNF) agents; (3) individuals with normal liver and kidney functions; and (4) those with no risk of tuberculosis activation or reactivation. The exclusion criteria were as follows: (1) patients with CD who were treated with biotherapy within the past 8 weeks, (2) active HIV or hepatitis B or C infection, (3) a history of surgery or trauma within 6 weeks, (4) allergy to cow or pig products, (5) elevated serum liver enzyme levels [levels of alanine aminotransferase (ALT) and aspartate aminotransferase (AST) over 40 U/L are defined as elevated], (6) elevated serum bilirubin levels [levels of total bilirubin (TBILI) over 24 µmol/L are defined as elevated], and (7) active malignant tumors within 5 years. None of the patients had taken antibiotics for at least 2 months before enrolment. Adverse events or serious adverse events were documented throughout the study. The results of routine blood tests and liver function and renal function tests were examined at baseline and at the end of each MSC infusions. Adverse events were evaluated at each follow-up visit. The stature, weight, body mass index (BMI), erythrocyte sedimentation rate (ESR), C-reactive protein (CRP) level, and CDAI scores were determined at baseline and after 4 and 8 MSC treatments. Colonoscopies were performed before MSC therapy and 3 months after the last MSC infusion. Mucosal inflammation was assessed using the Simple Endoscopic Score (SES-CD).

### Long-Term Follow-Up

Patients were followed up for 3 years after the last infusion of MSCs to diagnose and record possible adverse events and to monitor clinical efficacy. Patients were unable to return for follow-up visits and those who moved to different centers were contacted by telephone or WeChat to collect data on symptom recurrence, interval medications, surgery, hospitalization, and laboratory tests. Luminal Crohn’s disease relapse is assessed by lower endoscopy.

### Preparation, Culture, Identification, and Infusions of MSCs

The preparation of MSCs was approved by the Ethics Committee of the Third Affiliated Hospital of Sun Yat-sen University. The aseptic conditions for cell preparation and culture were standardized and maintained according to good manufacturing practice (GMP) at the Stem Cell Laboratory Facility of the Biotherapy Center at our hospital. MSCs were isolated and expanded from human umbilical cords using a previously reported protocol.^27^ In brief, the UC was dissected, and the umbilical vein and arteries were removed manually. The UC tissues and umbilical cord colloids were chopped into pieces with a diameter of approximately 2 mm and cultured in L-DMEM (Gibco). After cell separation, the cells were cultured in low-sugar Dulbecco’s modified Eagle’s medium (1 g/L DMEM, Gibco, Life). The medium contained 10% fetal bovine serum (FBS, Gibco, Life), the culture temperature was 37 °C, the air contained 5% CO_2_, and the humidity was 5%. Nonadherent cells were removed every 3 days by refreshing the medium. A flow cytometry analysis of CD90, CD19, CD11b, HLA-DR, CD34, CD45, CD105, and CD73 (Beckman Coulter, Brea) was performed to assess the phenotype based on cell surface marker expression. The differentiation potential was determined in accordance with criteria established by the 2006 International Society of Cellular Therapy, which examines the ability of MSCs to differentiate into osteocytes and adipocytes.^28^ MSCs at 70%-80% confluence were treated with trypsin. MSCs in passages 3-5 were collected and used in clinical trials. Tests confirmed that the cells were negative for HBV, HCV, HIV, fungi, syphilis, mycoplasma, and endotoxins before infusion.

Patients with CD received infusions of 1.0 × 10^6^ cells/kg hUC-MSCs (human umbilical cord-derived mesenchymal stem cells) through the peripheral vein. The first 4 infusions were administered at weekly intervals, and the last 4 infusions were administered at monthly intervals, resulting in a total of eight infusions.

### 16S rRNA Sequencing and Data Analysis

Fresh fecal samples were collected from the patients at baseline and after 4 and 8 rounds of MSC treatment. After defecation, fecal samples were collected, immediately placed on dry ice, and then transferred to the laboratory within 2 h, followed by storage at −80 °C until DNA extraction. DNA was extracted using a QIAamp Fast DNA Stool Mini Kit (Qiagen). The concentration of bacterial DNA was measured using a NanoDrop 2000 spectrophotometer (Thermo Fisher Scientific). Anthropometric data were collected during clinic visits.

The V3--V4 region of the bacterial 16S ribosomal RNA (rRNA) gene was amplified by PCR with barcode-indexed primers (338F and 806R) using FastPfu Polymerase. Amplicons were then purified by gel extraction (AxyPrep DNA Gel Extraction Kit, Axygen Biosciences) and quantified using a QuantiFluor-ST (Promega). Purified amplicons were pooled in equimolar amounts and paired-end sequenced on the NovaSeqPE250 platform (Illumina) according to the standard protocols provided by Guangdong Magigene Biotechnology Co., Ltd. (Guangzhou). The raw 16S rRNA gene sequencing reads were demultiplexed, quality filtered using fastp version 0.20.0,^29^ and merged using FLASH version 1.2.7.^30^

### Liquid Chromatography-Mass Spectrometry (LC-MS) Analysis

Fifty milligrams of fecal samples were removed from each patient with CD. This analysis was conducted using the same fecal samples as those used for 16S rRNA sequencing. The metabolites were extracted in a 400 µL solution composed of methanol:water (4:1, v/v). The mixture was incubated at −20 °C and then ground using a high-throughput tissue crusher (Wonbio-96c, Shanghai Wanbo Biotechnology Co., Ltd.) at 50 Hz for 6 min, followed by vortexing for 30 s and ultrasonication at 40 kHz for 30 min at 5 °C. The samples were placed at −20°C for 30 min to precipitate proteins. After centrifugation at 13 000×*g* and 4 °C for 15 min, the supernatants were carefully transferred into sample vials for LC--MS analysis.

The supernatants of each sample were transferred and mixed as a quality control sample. An AUHPLC-Q Exactive system was used for LC--MS detection. The chromatographic conditions were as follows: an ACQUITY UPLC HSS T3 chromatography column (100 mm × 2.1 mm i.d., 1.8 μm; Waters, Milford) was used; mobile phase A was composed of 95% water + 5% acetonitrile (including 0.1% HCOOH); mobile phase B was composed of 47.5% acetonitrile + 47.5% isopropanol + 5% water (including 0.1% HCOOH); the flow rate was 0.40 mL/min; the injection volume was 2 μL; and the chromatographic column temperature was 40 °C. Finally, Progenesis QI metabonomics software was used to extract, align, and identify the raw data.

### Statistical and Bioinformatics Analyses

Descriptive attributes that were assessed for statistical significance included age, stature, weight, BMI, ESR, CRP level, the proportion of female patients, and CDAI score, which are presented as the means and SDs. Groups were compared using repeated-measures analysis of variance (ANOVA) with Bonferroni’s post hoc analysis. Operational taxonomic units (OTUs) with a 97% similarity cut-off^31^ were clustered using UPARSE version 7.1,^32^ and chimeric sequences were identified and removed. The taxonomy of each OTU representative sequence was analyzed using RDP Classifier version 2.2^33^ and compared against the 16S rRNA database using a confidence threshold of 0.7. The observed species, Shannon, Simpson, and Chao1 indexes were used to evaluate the complexity of species diversity. By performing linear discriminant analysis effect size (LEfSe), we identified the features with the greatest contributions to the variation between the control and treatment groups [linear discriminant analysis (LDA) > 4]. The clinical indicators and the relative abundances of genera were analyzed using Spearman’s rank correlation analysis to obtain the corresponding correlation coefficient (Corr) matrix and correlation *P* value matrix. The multivariate statistical analysis was performed using the ropls (version 1.6.2, http://bioconductor.org/packages/release/bioc/html/ropls.html) R package from Bioconductor on the Majorbio Cloud Platform (https://cloud.majorbio.com). Principal component analysis (PCA) using an unsupervised method was applied to obtain an overview of the metabolic data, and general clusters, trends, or outliers were visualized. Partial least squares-based discriminant analysis (PLS-DA) was used for the statistical analysis to determine global metabolic changes between comparable groups. All metabolite variables were subjected to Pareto scaling prior to conducting PLS-DA. The model validity was evaluated by calculating the model parameters *R*^2^ and *Q*^2^, which provide information on the interpretability and predictability, respectively, of the model and prevent the risk of overfitting. The variable importance in projection (VIP) was calculated in the PLS-DA model. *P* values were estimated with paired Student’s *t*-test or single-dimensional statistical analysis.

Statistically significant differences among groups were selected with a VIP value greater than 1 and a *P* value less than .05. Differentially abundant metabolites among the 2 groups were summarized and mapped into their biochemical pathways through metabolic enrichment and pathway analyses by searching a database (KEGG, http://www.genome.jp/kegg/). These metabolites were classified according to the pathways in which they are involved or the functions they perform. An enrichment analysis usually assesses a group of metabolites in a functional node to determine whether they appear. The principle was that the analysis of the annotation of a single metabolite develops into an analysis of the annotations of a group of metabolites. The scipy.stats Python package (https://docs.scipy.org/doc/scipy/) was exploited to identify significantly enriched pathways using Fisher’s exact test.

The levels of fecal metabolites and the relative abundances of genera were analyzed by calculating Pearson’s correlation coefficients to obtain the corresponding correlation coefficient (Corr) matrix and correlation *P* value matrix. We determined the correlations between only those genera and metabolites for which *P* < .05. In all statistical tests, *P* < .05 was considered significant.

## Results

### Clinical Characteristics of Patients with CD

Eight patients with CD (mean age, 36.1 ± 7.2 years) were enrolled in this study. The baseline characteristics are presented in Table 1. The proportion of female patients was 62.5%. After 8 infusions of MSCs, 6 patients provided 3 stool samples each. One patient received only 4 MSC infusions and refused further clinical treatment. This patient withdrew from the study without giving any reasons. One patient was infused with 8 rounds of MSCs; however, no stool samples were collected at the last time point due to constipation.

**Table 1.** Baseline characteristics of the enrolled patients.

Patient number	#1	#2	#3	#4	#5	#6	#7	#8
Age (years)	32	34	31	37	48	36	45	26
Sex	F	M	F	M	F	F	F	M
Disease duration (years)	5	13	2	2	12	10	7	2
Smoking status	n	n	n	n	n	n	n	n
Prior medical therapies	5-ASA, AZA, CSs	TPT, AZA, MTX, CSs	IFX, MTX	5-ASA, CSs, AZA	AZA, CSs, 5-ASA,	AZA, TPT, CSs	MTX, IFX, AZA, TPT	AZA, TPT, 5-ASA
Prior surgeries	n	Colonic resection	n	n	Ileocecal resection	n	n	Right hemicolectomy,
Current medical therapies	AZA	AZA, TPT	n	AZA	AZA	AZA, TPT	n	AZA, TPT
Disease localization	Ileocolonic	Colonic	Colonic	Ileocolonic	Ileocolonic	Ileocolonic	Ileocolonic	Colonic
Natural history of perianal disease	y (anal fistula)	n	y (perianal abscess)	n	y (anal fistula)	n	y (perianal abscess)	n
Height (cm)	155	159	160.5	166.5	153	159.5	159	165
Weight (kg)	44.4	62.9	46	62.5	42.8	48.5	53.3	47.8
Baseline CDAI	175.4	243.5	329	239.2	244.1	202.7	235.7	261.5
No. of MSCs infusions received	8	8	4	8	8	8	8	8

Abbreviations: 5-ASA, Mesalamine; AZA, azathioprine; CSs, corticosteroids; TPT, thalidomide; MTX, methotrexate; IFX, infliximab; F, female; M, male; n, no; y, yes; CDAI, Crohn’s disease activity index.

### Safety

Two patients experienced fever after the first UC-MSC infusion, which was relieved without treatment. No serious adverse effects were observed. None of the patients displayed dyspnea following the infusion. The red blood cell count, liver function, and renal function were all normal.

### Short-Term Efficacy Outcomes

Overall, the clinical symptoms and signs of the patients with CD were substantially relieved after 8 MSC infusions, as revealed by changes in the weight, BMI, CDAI score, CRP levels, and ESR (Supplementary Table S1). The data of the participants are described in Table 1. The weight of the patients increased from 51.0 ± 7.8 kg to 52.4 ± 8.0 kg, and 55.0 ± 9.1 kg after 4 and 8 rounds of MSC treatment, respectively (*F* = 19.372, *P* < .001). The BMI increased from 20.0 ± 2.6 kg/m^2^ to 20.5 ± 2.7 kg/m^2^ and 21.5 ± 3.0 kg/m^2^ after 4 and 8 rounds of MSC treatment, respectively, and these increases were not significant (*F* = 0.371, *P* = .697). The ESR decreased from 19.1 ± 15.0 mm/h to 17.6 ± 15.5 mm/h and 14.5 ± 11.1 mm/h after 4 and 8 rounds of MSC treatment, respectively (*F* = 4.571, *P* < .05). The CRP level decreased from 12.2 ± 10.7 mg/L to 8.1 ± 9.1 mg/L and 3.1 ± 2.0 mg/L after 4 and 8 rounds of MSC treatment, respectively (*F* = 5.424, *P* < .05). The CDAI score decreased from 228.9 ± 29.5 to 193.1 ± 30.2 and 150.1 ± 36.7 after 4 and 8 rounds of MSC treatment, respectively (*F* = 28.694, *P* < .001).

**Figure 1.**
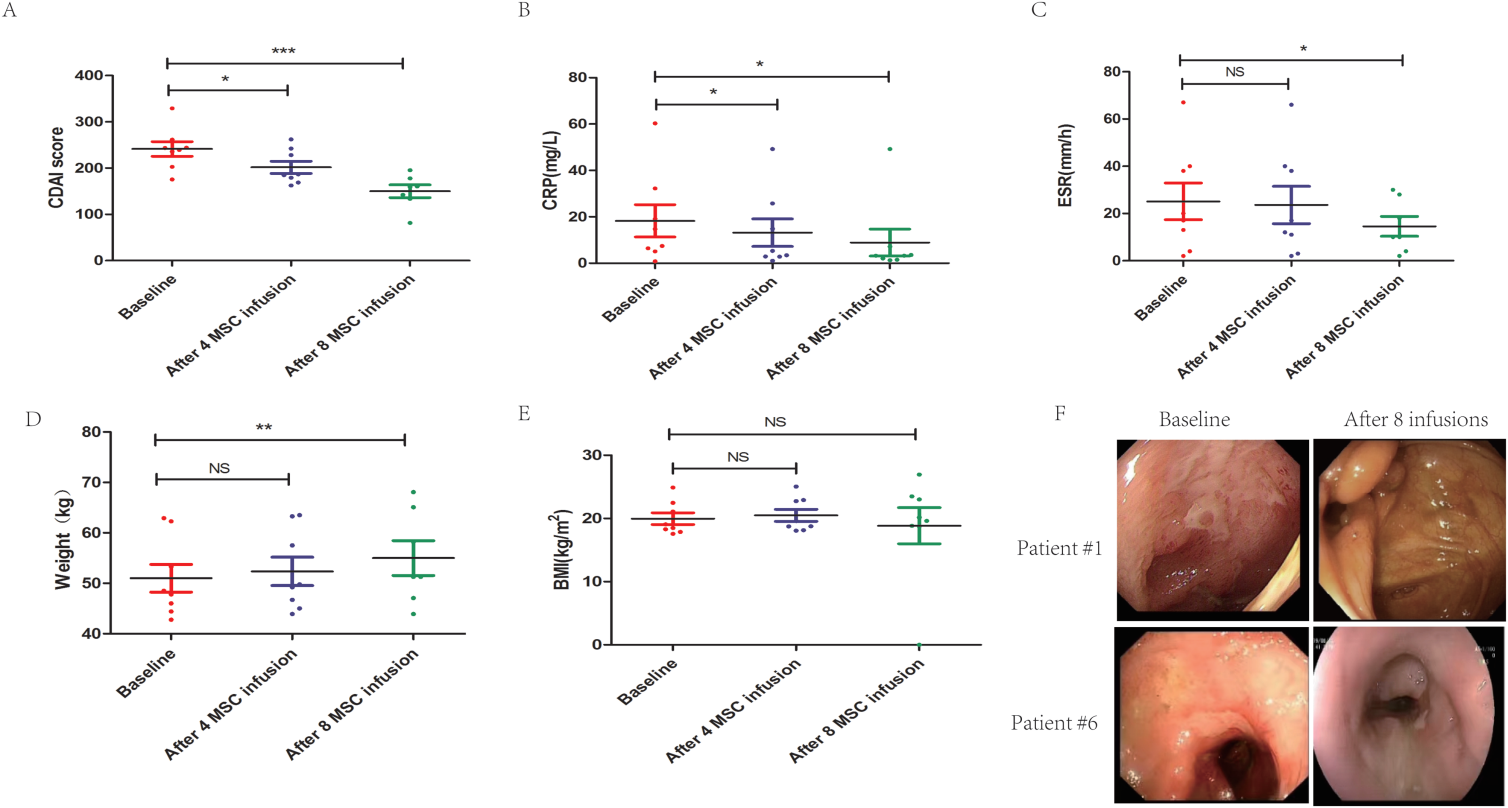
Comparisons of changes in clinical indices and biological data before and after MSC infusions. (**A**) Crohn’s disease activity index (CDAI). (**B**) C-reactive protein (CRP). (**C**) Erythrocyte sedimentation rate (ESR). (**D**) Weight. (**E**) Body mass index (BMI). The Bonferroni test was used for multiple comparisons: *P < .05, **P < .01, and ***P < .001. (**F**) Endoscopy at baseline (left panels) and after 8 mesenchymal stromal cell (MSC) infusions (right panels) shows clear mucosal healing in patients 1 (upper panels) and 6 (lower panels). Mucosal inflammation was assessed by the Simple Endoscopic Score (SES-CD).

Compared with the values recorded at baseline, the CDAI score (Fig. 1A) and CRP level (Fig. 1B) decreased after 4 rounds of MSC treatment (*P* < .05). The CDAI score (*P* < .001) (Fig. 1A), CRP level (*P* < .05) (Fig. 1B), and ESR (*P* < .05) (Fig. 1C) decreased and the weight (*P* < .01) (Fig. 1D) increased after 8 rounds of MSC treatment. However, after 4 or 8 rounds of MSC infusion, no significant increase in the BMI compared with the baseline was detected (Fig. 1E). The SES-CD decreased from 8 to 4 after 8 MSC infusions in patient 1 and from 23 to 11 after 8 MSC infusions in patient #6. Endoscopic improvement was observed in these 2 patients (Fig. 1F). In 3 other patients (patients #2, #3, and #5), no significant endoscopic improvement was observed between baseline and 3 months after the last MSC infusions. Patients #4, #7, and #8 declined endoscopic evaluation 3 months after the last MSC infusion without giving any reasons.

### Long-Term Efficacy Outcomes

Patient #3 received only 4 MSC infusions and refused further clinical treatment and long-term follow-up. There were 7 patients (median age, 39 years old; range: 29-51 years old) who completed the 8 rounds of MSC therapy that could be evaluated during the subsequent three years. After 8 rounds of MSC treatment, significant improvement was observed. As shown in Supplementary Table S2, patient #1 had a relapse of anal fistula after 24 months, followed by biological therapy with infliximab. Patient #2 had a relapse of diarrhea and hematochezia after 21 months, followed by biological therapy with infliximab, resulting in disease control and general well-being. Patient #4 was in stable condition. Patient #5 had a relapse of perianal abscess after 3 months, followed by biological therapy with infliximab, resulting in stable disease. Patient #6 had weight loss and an increase in the number of ileocolonic ulcers as determined by endoscopy after 27 months followed by biological therapy with vedolizumab, resulting in disease control and general well-being. Patient #7 had a relapse of perianal abscess after 3 months followed by biological therapy with vedolizumab. Patient #7 used vedolizumab twice in 6 weeks with worsening symptoms of perianal abscess. Patient #7 switched to ustekinumab, symptoms resolved, and the condition stabilized. Patient #8 developed intestinal obstruction 23 months after the last MSC infusion. Following colectomy, treatment with vedolizumab was administered, and the condition was stable.

### Changes in the Fecal Microbial Diversity

**Figure 2.**
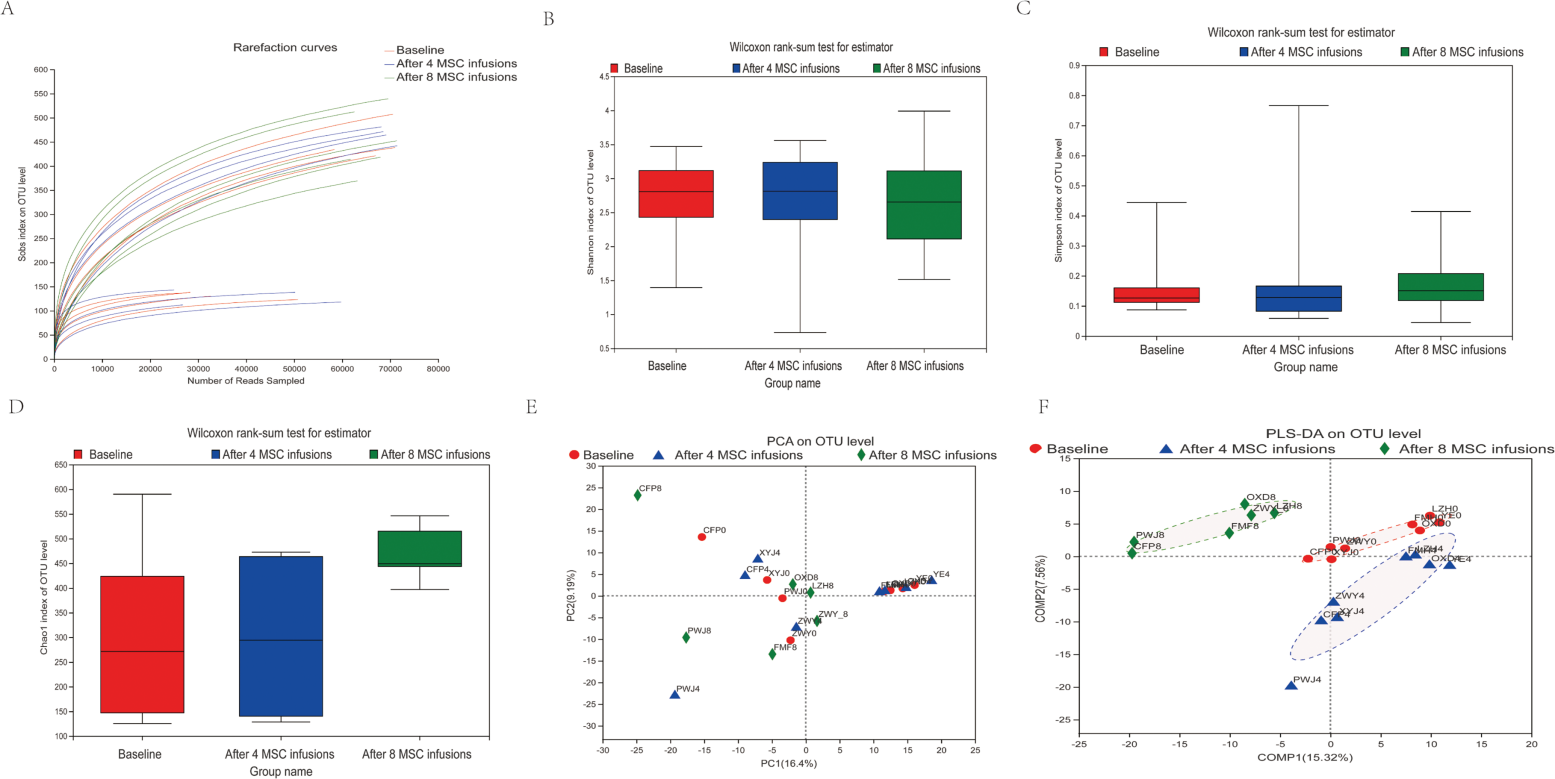
Changes in the fecal microbial diversity of patients with Crohn’s disease before and after 4 and 8 MSC infusions (*n* = 8 for the 4 MSC infusion group and *n* = 6 for the 8 MSC infusion group). (**A**) The curve of each sample was nearly smooth, indicating that the sequencing data were sufficient. (**B-D**) The Shannon, Simpson, and Chao1 indexes were calculated to estimate the diversity of the fecal microbiota among the 3 groups. (**E**) PCA at the operational taxonomic unit (OTU) level. (**F**) PLS-DA based on the abundance of OTUs.

The microbial diversity was measured and compared at the OTU level. The rarefaction curve reached stable values in the current sequencing analysis (Fig. 2A). This result indicated that the sequencing depth covered rare, new phylotypes. We compared the α-diversity of the microbiota between the baseline and after 4 or 8 MSC interventions by calculating the Shannon, Simpson, and Chao1 indexes. Compared with the results obtained at baseline, no significant differences in the α-diversity (Shannon, Simpson, and Chao1 indexes) were observed after 4 (*P* = .958, *P* = .7929, and *P* = .9581, respectively) or 8 (*P* = .9485, *P* = .6514, and *P* = .0813, respectively) MSC interventions (Fig. 2B-2D). PCA was conducted at the OTU level to evaluate the extent of bacterial community similarity and showed no obvious differences between bacterial groups (*R* = −0.0285, *P* = .703000) (Fig. 2E). PLS-DA is a chemometrics technique used to optimize separation between different groups of samples.^34^ PLS-DA at the OTU level showed separate clusters, suggesting that the overall structures of the bacterial communities in the groups were significantly different (Fig. 2F).

**Figure 3.**
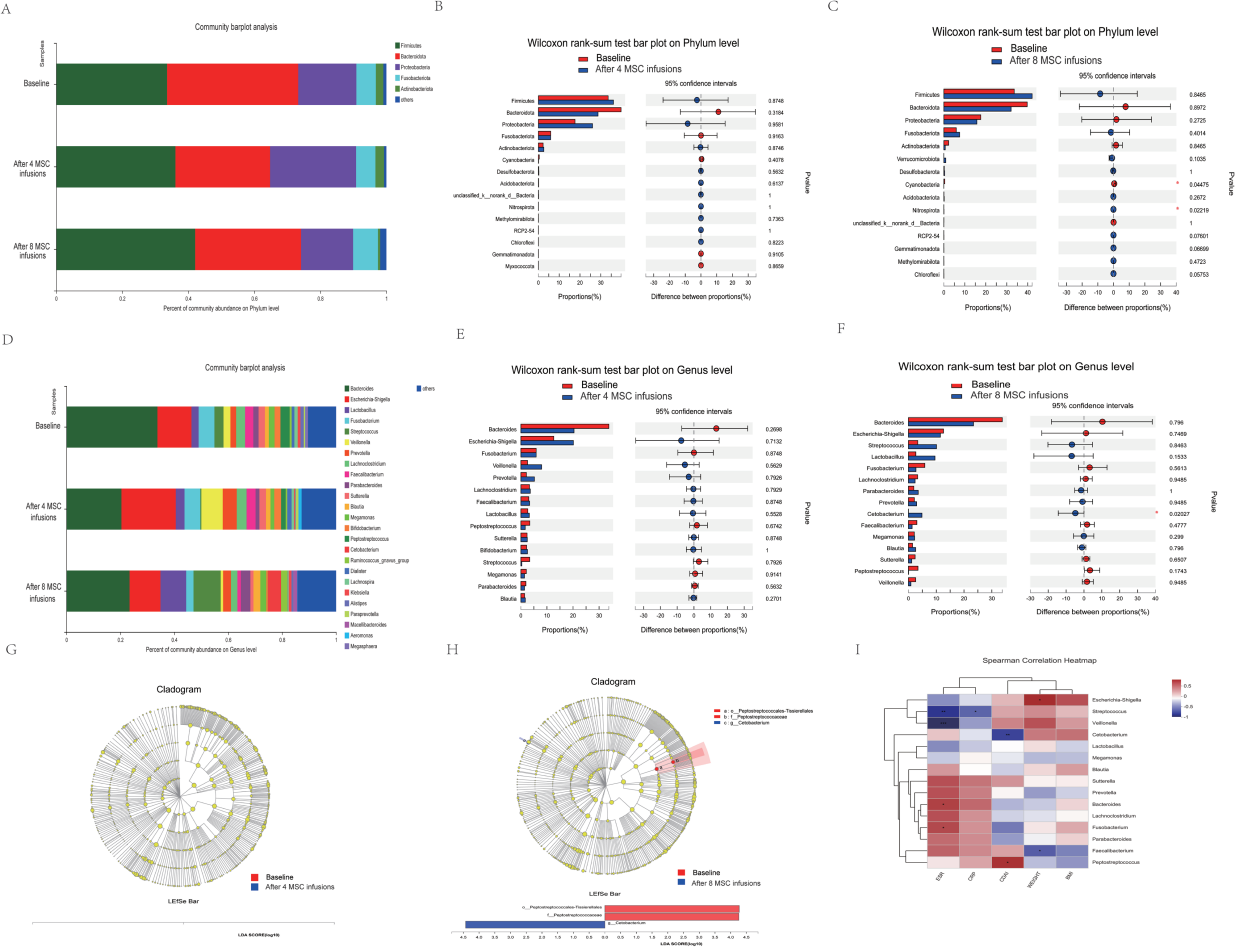
Microbiota composition was determined by 16S rDNA sequencing in patients with CD before and after 4 and 8 MSC infusions (*n* = 8 for the 4 MSC infusion group and *n* = 6 for the 8 MSC infusion group). (**A**) Relative abundance of predominant bacteria at the phylum level. (**B**) Bar plot of the Wilcoxon rank-sum test at the phylum level before and after 4 MSC infusions. (**C**) Bar plot of the Wilcoxon rank-sum test bar at the phylum level before and after 8 MSC infusions. (**D**) Relative abundance of predominant bacteria at the genus level. (**E**) Bar plot of the Wilcoxon rank-sum test at the genus level before and after 4 MSC infusions. (**F**) Bar plot of the Wilcoxon rank-sum test at the genus level before and after 8 MSC infusions. (**G**) LEfSe was performed to distinguish the differentially abundant microbiome constituents before and after 4 MSC infusions. Differently colored nodes indicate populations of bacteria that were significantly enriched in the corresponding populations and that showed differences between them. Yellow nodes indicate no significant differences between microbial groups. LDA was conducted, and only microbiota constituents with scores greater than 4 are shown. (**H**) LEfSe was performed to distinguish the differentially abundant microbiome constituents before and after 8 MSC infusions. LDA was conducted, and only microbiota constituents with scores greater than 4 are shown. (**I**) Spearman’s rank correlation analysis of the top 15 enriched bacterial genera and clinical indicators. The heatmap shows that microbial taxa were intimately associated with clinical indicators. Colors represent the Spearman rank correlation coefficients. *P < .05 and **P < .01 denote statistically significant differences between bacterial taxa and clinical indicators.

A detailed description of the changes in the intestinal microbiota is provided in Fig. 3A (phylum level) and D (genus level). The results obtained at baseline and the 4 or 8 MSC intervention time points at the phylum level are shown in Fig. 3B, 3C. The cyanobacteria phylum was depleted, and the nitrospirota phylum was enriched after 8 MSC infusions (*P* < .05, Wilcoxon rank-sum test; Fig. 3C). The results obtained for the genus level at baseline and 4 or 8 MSC intervention time points are shown in Fig. 3E, 3F. The *Cetobacterium* genus was enriched after 8 MSC infusions (*P* < .05, Wilcoxon rank-sum test; Fig. 3F). At the phylum and genus levels, statistically significant differences in microbial abundances were not detected at the baseline and 4 MSC intervention time points (Fig. 3B, 3E).

In the comparisons using LEfSe, a significant difference in specific components of the gut microbiota was not observed between baseline and 4 MSC interventions (Fig. 3G). Nevertheless, cyanobacteria were depleted at the phylum level, and the *Cetobacterium* genus was significantly enriched after 8 rounds of MSC intervention compared to that at baseline (Fig. 3H). As shown in Fig. 3I, Spearman’s correlation analysis indicated that the changes in weight were positively associated with the changes in *Escherichia-Shigella* abundance (*r* = 0.65787, *P* = .01055) and negatively associated with the changes in *Faecalibacterium* abundance (*r* = −0.56326, *P* = .03596). The changes in the CDAI score were positively associated with the changes in *Peptostreptococcus* abundance (*r* = 0.63436, *P* = .01482) and negatively associated with the changes in *Cetobacterium* abundance (*r* = −0.66164, *P* = .00996). The changes in CRP levels were negatively associated with the changes in *Streptococcus* abundance (*r* = −0.53744, *P* = .04747). The changes in the ESR were positively associated with the changes in *Bacteroides* (*r* = 0.57837, *P* = .03026) and *Fusobacterium* abundance (*r* = .54907, *P* = .042) and were negatively associated with the changes in *Streptococcus* (*r* = −0.70861, *P* = .00456) and *Veillonella* abundance (*r* = −0.81809, *P* = .00035).

### Effects of MSCs on Fecal Metabolites

**Figure 4.**
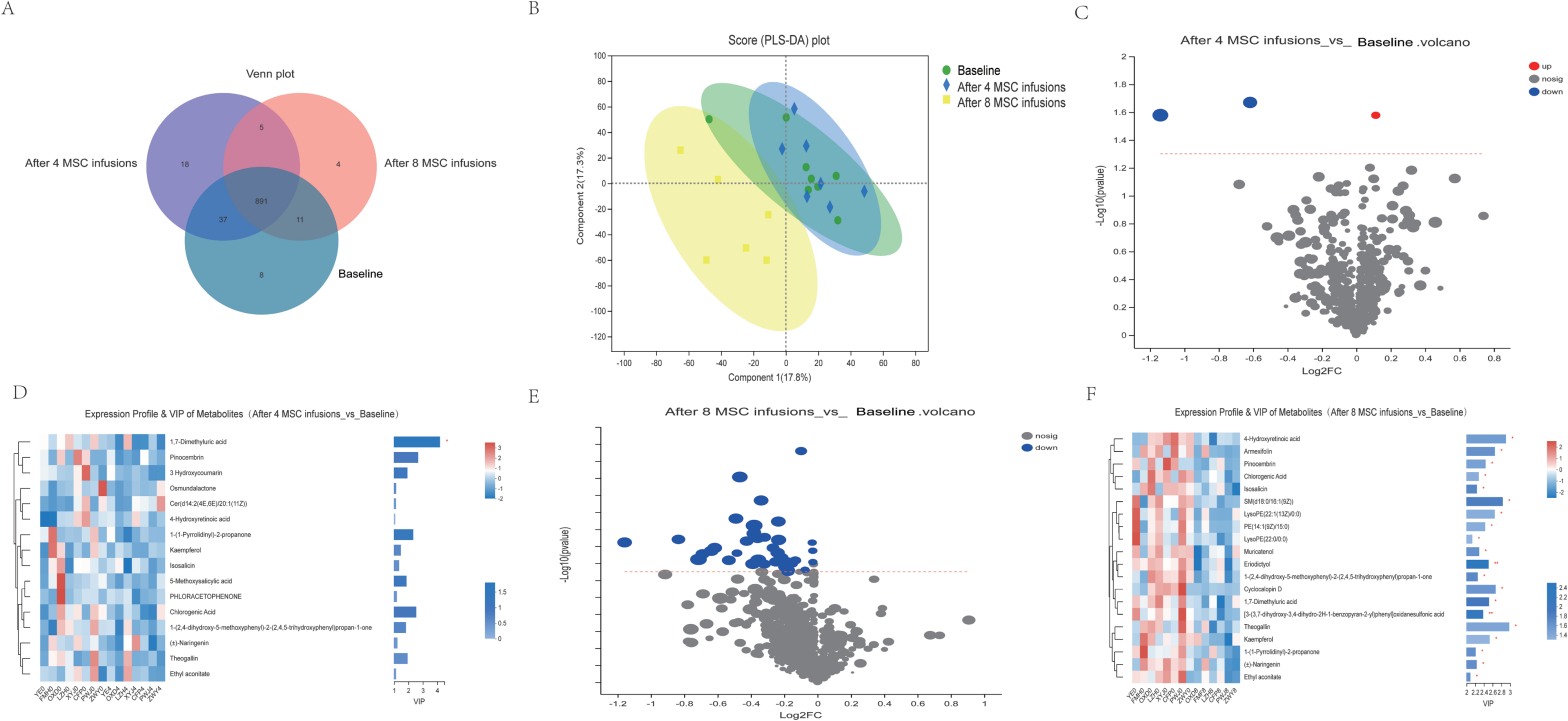
Changes in the fecal metabolome after 4 and 8 MSC infusions (*n* = 8 for the 4 MSC infusion group and *n* = 6 for the 8 MSC infusion group). (**A**) Venn diagram of differentially abundant metabolites before the MSC infusion (blue circles) compared with after 4 MSC infusions (purple circles) and 8 MSC infusions (pink circles). (**B**) PLS-DA score plot of metabolites before the MSC infusion (green circle), after 4 MSC infusions (blue diamond), and after 8 MSC infusions (yellow square). (**C**) A volcano plot was drawn to show the distribution of all metabolites identified using LC--MS before the MSC infusion and after 4 MSC infusions. (**D**) Heatmap of the enrichment and depletion of 1 differentially abundant metabolite with VIP > 1 and *P* < .05 identified using LC--MS after 4 rounds of MSC infusions (*P < .05 and **P < .01). (**E**) A volcano plot was drawn to show the distribution of all metabolites identified using LC--MS before the MSC infusion and after 8 rounds of MSC infusions. (**F**) Heatmap of the enrichment and depletion of 30 differentially abundant metabolites with VIP > 1 and *P* < .05 identified using LC--MS after 8 rounds of MSC infusions (*P < .05 and **P < .01).

Fecal metabolites were quantified in all fecal samples using LC--MS to determine the intestinal metabolites that were involved in the MSC-induced alleviation of CD. We detected 888 metabolites, of which 733 were detected in the metabolite library and 279 were detected in the KEGG database. The 974 metabolites included 947 metabolites detected at baseline, 951 metabolites detected after 4 MSC interventions, and 911 metabolites detected after 8 MSC interventions. Forty-two significantly differentially abundant metabolites were identified between baseline and 4 MSC infusions, 54 between baseline and 8 MSC infusions, and 22 between 4 MSC infusions and 8 MSC infusions (Fig. 4A). PLS-DA was subsequently performed to distinguish the differences in metabolite levels at different time points (baseline and after 4 and 8 MSC interventions), and the score plot of the PLS-DA indicated that baseline and 8 MSC interventions were obviously distinguished (Fig. 4B). Metabolites detected at baseline and after 8 MSC infusions were separated. Fig. 4C, 4D reveals the alterations in the metabolite levels after 4 MSC infusions. As a result, the 4 MSC infusion groups showed significantly lower levels of 1,7-dimethyluric acid (VIP > 1 and *P* < .05; Fig. 4D). Fig. 4E and F reveals the alterations in the metabolite levels after 8 MSC infusions. As a result, the 8 MSC infusion group showed significantly lower levels of 30 differentially abundant metabolites (VIP > 1 and *P* < .05).

**Figure 5.**
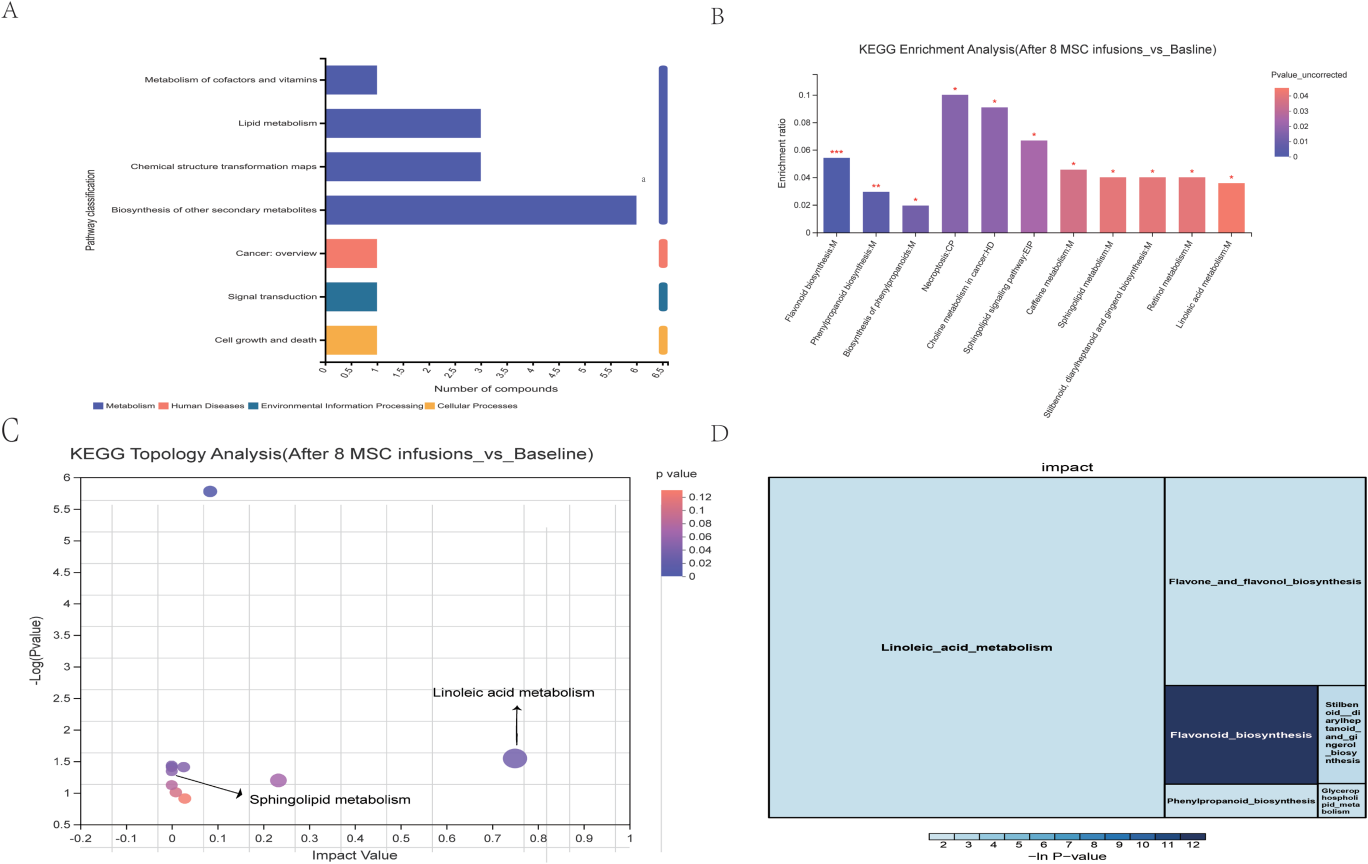
KEGG pathway analysis. (**A**) KEGG second-level functional distribution (after 8 MSC infusions vs. before the MSC infusion). The *x*-axis represents the second-level terms in the KEGG pathway, and the *y*-axis represents the number of metabolites identified. (**B**) Analysis of enriched metabolic pathways for differentially abundant metabolites between baseline and after 8 MSC infusions. The *X*-axis represents the significant enrichment of the participating metabolite pathway. The *Y*-axis represents the enrichment ratio, indicating the ratio of the number of metabolites enriched in the pathway and the number of metabolites identified in the pathway. The column color represents the significance of the enrichment. The deeper the color, the more significant the enrichment of the KEGG term (*P < .05, **P < .01, and ***P < .001). M, metabolism; CP, cellular process; HD, human disease; EIP, environmental information processing. (**C**) Topological analysis of metabolic pathways between baseline and after 8 MSC infusions. The *x*-axis represents the relative importance of metabolites in the pathway and the size of the impact value. The *y*-axis represents the significance of metabolites enriched (−log10 (*P* value)) in the pathway; the bubble size represents the impact value. The bubble color represents the *P* value. The larger the bubble is, the greater the importance of the pathway. (**D**) KEGG rectangular tree diagram showing the size of the impact and enrichment of metabolic pathways by color and square size, enhancing metabolic pathway identification by integrating enrichment and topology analyses.

The metabolites identified above were assigned to the KEGG database. Metabolic pathway enrichment and topological analyses of the metabolic pathways were performed using the KEGG metabolic pathway database. For the “metabolism” term, the top priority was “biosynthesis of other secondary metabolites,” followed by “lipid metabolism,” “chemical structure transformation maps,” and “metabolism of cofactors and vitamins” (Fig. 5A). Five metabolites (kaempferol, pinocembrin, chlorogenic acid, eriodictyol, and 1,7-dimethyluric acid) were assigned to “biosynthesis of other secondary metabolites.” Three metabolites (linoleic acid, SM(d18:0/16:1(9Z)), and lysoPC(24:0)) were assigned to “lipid metabolism.” Two metabolites (linoleic acid and kaempferol) were assigned to “chemical structure transformation maps,” and 1 metabolite (4-hydroxyretinoic acid) was assigned to “metabolism of cofactors and vitamins.”

An analysis of metabolite pathways suggested that the following pathways were significantly changed after 8 MSC infusions compared with those at baseline: flavonoid biosynthesis, phenylpropanoid biosynthesis, biosynthesis of phenylpropanoids, necroptosis, choline metabolism in cancer, sphingolipid signaling pathway, caffeine metabolism, sphingolipid metabolism, glutenin, diarylheptanoid and gingerol biosynthesis, retinol metabolism, and linoleic acid metabolism (Fig. 5B). A topological analysis of the pathways was performed to determine the impact of the pathway based on the size of the bubble (Fig. 5C). A KEGG rectangular tree diagram was generated using the impact and enrichment of the metabolic pathway based on the size and color of the square (Fig. 5D). Linoleic acid metabolism was the main pathway altered between baseline and 8 MSC treatments (Fig. 5C, 5D).

### Correlation Between Fecal Metabolites and the Microbiota

**Figure 6.**
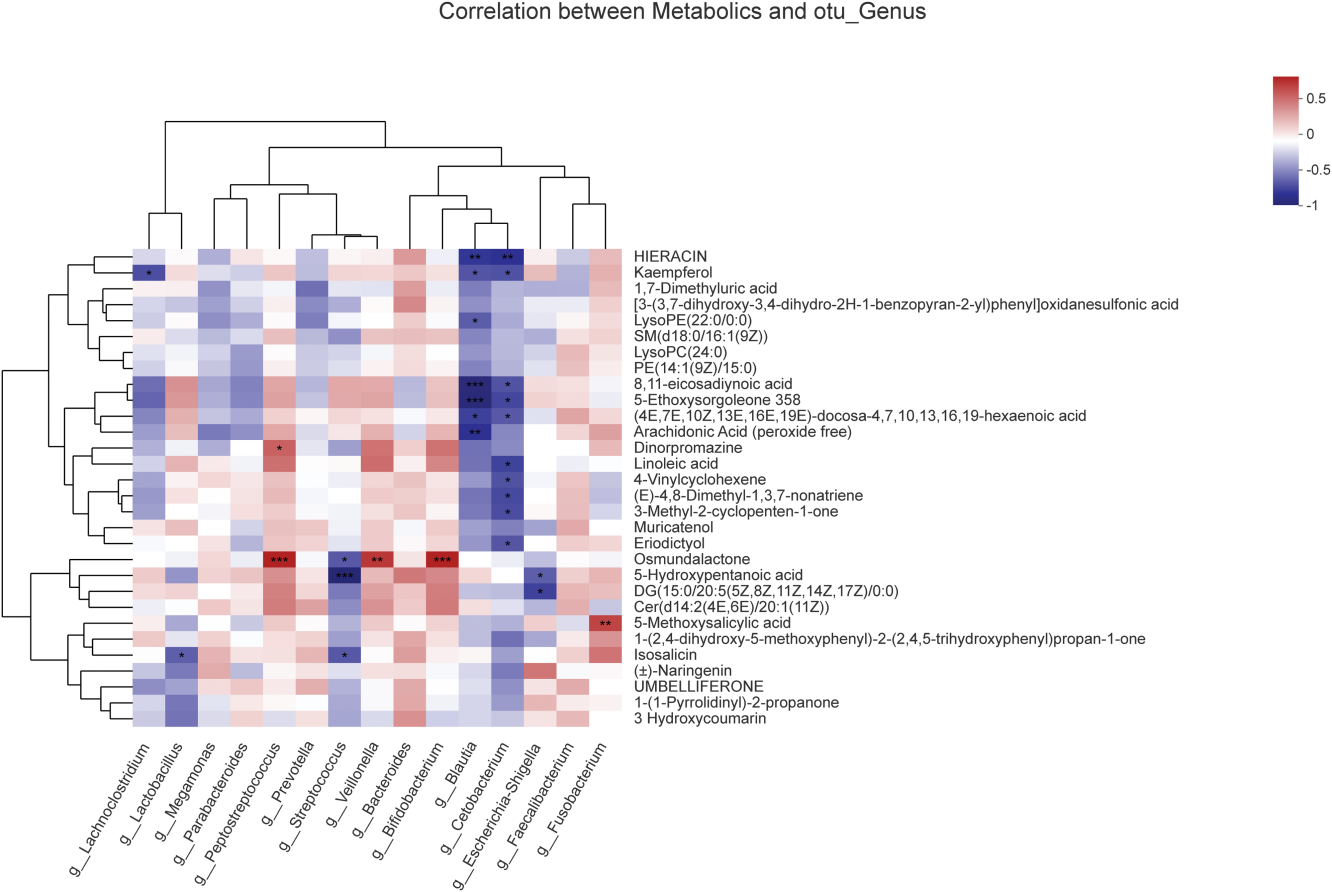
Relationship between changes in fecal metabolite levels and genus abundances. The heatmap illustrates the Pearson correlation coefficients between changes in fecal metabolite concentrations and changes in the relative abundances of the top 15 enriched bacterial genera. The change in the relative abundance of individual genera is shown; the intensity of the colors represents the degree to which changes in fecal metabolite levels are correlated with changes in relative abundance. *P* values were corrected for multiple testing using the Benjamini--Hochberg false discovery rate: *P < .05 and **P < .01.

The Pearson correlation coefficients between the levels of selected metabolites and the relative abundances of fecal microbiota constituents at the genus level were calculated. A significant correlation between the fecal metabolites and abundance of genera in the fecal microbiota was observed after 8 MSC infusions. As shown in Fig. 6, Pearson’s correlation analysis revealed that the change in the abundance of the genus *Cetobacterium* was negatively associated with the changes in the levels of linoleic acid (*r* = −0.6477, *P* = .01225), (4E,7E,10Z,13E,16E,19E)-docosa-4,7,10,13,16,19-hexaenoic acid (*r* = −0.5695, *P* = .0335), 3-methyl-2-cyclopenten-1-one (*r* = −0.611, *P* = .02027), 4-vinylcyclohexene (*r* = −0.5904, *P* = .02623), (*E*)-4,8-dimethyl-1,3,7-nonatriene (*r* = −0.6223, *P* = .01747), 3-methyl-2-cyclopenten-1-one (*r* = −0.611, *P* = .02027), 8,11-eicosadiynoic acid (*r* = −0.5871, *P* = .0273), 5-ethoxysorgoleone 358 (*r* = −0.6336, *P* = .01498), hieracin (*r* = −0.7199, *P* = .00369), kaempferol (*r* = −0.5943, *P* = .02501), and eriodictyol (*r* = −0.5775, *P* = .03058).

## Discussion

Our study is the first to document short-term changes in the gut microbiota in patients with relapsing refractory CD after MSC treatment. First, we focused on the safety and efficacy of MSC treatment. No serious adverse effects (SAEs) were observed. The CDAI score and CRP level decreased after 4 rounds of MSC treatment. The CDAI score, ESR, and CRP level decreased, while weight increased after 8 rounds of MSC treatment. Endoscopic improvement was observed in 2 patients. Furthermore, we applied a 16S rRNA sequencing approach and found that the *Cetobacterium* abundance increased after 8 MSC infusions compared to that at baseline. However, no significant differences in α-diversity were observed after 4 and 8 MSC infusions. In addition, fecal metabolites were quantified in all fecal samples using LC--MS to determine the effects of MSCs on fecal metabolite levels. Following 8 rounds of MSC treatment, 30 differentially abundant metabolites were present at significantly lower levels. Finally, we analyzed the correlations between fecal metabolites and the microbiota. Pearson’s correlation analysis showed that the change in *Cetobacterium* abundance was negatively correlated with the changes in the levels of linoleic acid, 4-vinylcyclohexene, 3-methyl-2-cyclopenten-1-one, 8,11-eicosadiynoic acid, 5-ethoxysorgoleone 358, hieracin, kaempferol, and eriodictyol.

The schedule, route of administration, and dose have varied in previous clinical trials. Duijvestein et al. infused bone marrow (BM)-MSCs into refractory luminal CD patients at a dose of 1-2 × 10^6^ cells/kg once a week for a total of 2 times.^35^ The injection schedule and dose in the study of Rachele Ciccocioppo et al. for treatment of fistulizing CD were used as follows: BM-MSCs were locally injected at intervals of 4 weeks, with a median of 20 × 10^6^ cells injected each time. When remission or improvement was achieved, or when autologous MSCs were no longer available, injections were stopped.^36^ Zhang et al. infused UC-MSCs into relapsing refractory CD patients at a dose of 1 × 10^6^ cells/kg once a week for a total of 4 times.^37^ Most of the patients in the study conducted by Zhang et al. used steroids during the infusion and follow-up periods. There is no consensus regarding the MSCs infusion schedule and dose, which are selected according to the severity of the patient’s condition. In our study, we enrolled relapsing refractory CD patients who failed to respond to corticosteroids, azathioprine, methotrexate, and TNF agents. The first 4 MSC (1 × 10^6^ cells/kg) infusions were administered at weekly intervals as was done in the study of Zhang et al. Additionally, patients received 4 other infusions of MSCs as consolidation therapy, and the interval was extended to 1 month.

Rigorous safety assessments of new therapies are necessary. Consistent with the present results, previous studies have reported a relatively high safety profile.^38^ In our study, no serious adverse reactions that required hospitalization were observed. Our trial indicated that MSCs are safe. Two of our patients experienced fever after MSC infusions. No SAEs were observed. None of the patients displayed dyspnea following the infusions.

We evaluated the efficacy of MSCs by comparing weight, BMI, CDAI score, CRP levels, and ESR before and after MSC treatment. After 4 and 8 rounds of MSC treatment, weight increased, while the CDAI score, CRP level, and ESR decreased. Similar to our data, Zhang et al. revealed that UC-MSCs reduced the CDAI score and were an effective treatment for CD.^37^ Endoscopic improvement was observed in 2 patients. In the other 2 patients, no significant endoscopic improvement was observed between baseline and after 8 MSC infusions. Similar to our studies, a proportion of patients with refractory CD do not achieve endoscopic improvement after autologous bone marrow-derived MSC therapy, but their clinical symptoms are relieved.^35^

The clinical symptoms and signs of patients with CD were significantly improved in the short term after 8 MSC infusions. Whether MSC infusions induce durable remissions worthy of further study. Our preliminary long-term efficacy results showed that almost all patients relapsed within 24 months, demonstrating that MSC infusions are far from a complete cure, but increase the remission rate in patients with refractory disease. Similar results were found by other scholars. Guadalajara et al. reported that only a small proportion of patients treated with stem cells remained relapse-free after more than 3 years of follow-up.^39^ Similarly, Ciccocioppo et al. showed that the mean CDAI score increased significantly within the second year and that most patients relapsed to varying degrees at later time points after local injection of MSCs.^40^

It is worth investigating the mechanisms by which the administered MSCs exert their effect. Investigators have conducted some research on the relationship between gut microbiota and stem cells in the treatment of CD. This research is of great importance in clarifying the mechanism of stem cell therapy for CD. The microbiota of mice with dextran sodium sulfate (DSS)-induced colitis was partially restored after treatment with MSCs.^41^ Our previously published animal experiments also indicated that MSC treatment could restore the microbiota function to normal in the TNBS mouse colitis model by modulating dysregulated metabolism pathways.^42^ This study has implications for understanding whether MSCs affect the gut microbiota in patients with CD. The research presented here confirms that patients were clinically stable and exhibited a slight amelioration of clinical symptoms, but no significant changes were observed in bacterial taxonomic richness, evenness or relative abundance at the phylum and genus levels after 4 rounds of MSC treatment. The majority of fecal metabolite levels were not significantly altered after 4 MSC treatments. However, the clinical symptoms were substantially alleviated after 8 rounds of MSC treatment. Comparing the results obtained after 8 MSC treatments with the baseline results, we found that the overall structures of the bacterial communities in the groups were significantly different. The *Cetobacterium* genus was significantly enriched after 8 MSC interventions compared to that at baseline.

*Cetobacterium somerae* has been characterized as a previously unknown microaerotolerant, gram-negative, and rod-shaped bacterium from feces collected from 2 children with autism, which was not present in these children prior to treatment with antibiotics.^43^ Vancomycin treatment is beneficial for regressive-onset autism related to abnormal gut microbiota.^44^ The patients showed a good response to vancomycin treatment at the time the organisms were isolated.^43^*C. somerae* administration to both adult and gnotobiotic larval zebrafish models improved glucose homeostasis and increased insulin levels.^45^*C. somerae* XMX-1 fermentation improves gut and liver health, as well as resistance to pathogenic bacteria in tilapia, and enhances antiviral immunity in zebrafish.^46,47^ Few previous reports have described the relationship between *Cetobacterium* and CD. Nishino et al. compared CD patients with non-IBD controls and found a significant increase in the genus *Cetobacterium.*^48^ Interestingly, we found that *Cetobacterium* was significantly enriched after 8 rounds of MSC intervention compared with that at baseline. After 8 MSC treatments, *Cetobacterium* was the only genus with a significant difference in abundance among the high-abundance genera. We identified correlations between clinical indicators and changes in the gut microbiome in patients with CD after 8 MSC treatments by performing a Spearman correlation analysis of altered gut microbiota and clinical indicators. We revealed that the CDAI score was strongly correlated with alterations in *Cetobacterium* abundance.

The primary pathway mediating the interaction between the gut microbiota and the host is metabolites, which are small molecules that are produced as end products or intermediates of microbial metabolism.^49^ The goal of metabolomics is to quantitatively describe the dynamic changes in various metabolites within organisms. Some specific metabolites may reflect the metabolic characteristics of an individual or disease.^50^ By applying an integrated metabolomics analysis of feces, we identified a number of metabolites in patients with CD who were treated with 8 rounds of MSCs. In fecal samples, the differentially abundant metabolites were mainly involved in flavonoid biosynthesis, phenylpropanoid biosynthesis, biosynthesis of phenylpropanoids, necroptosis, choline metabolism in cancer, sphingolipid signaling pathway, caffeine metabolism, sphingolipid metabolism, glutenin, diarylheptanoid and gingerol biosynthesis, retinol metabolism, and linoleic acid metabolism. The sphingolipid signaling pathway, sphingolipid metabolism, and linoleic acid metabolism are important metabolic pathways related to IBD.^51,52^ Dysregulated production of several sphingolipid molecules is associated with IBD and plays a major role in its pathogenesis and perpetuation.^53^ Sphingolipid molecules were significantly overabundant in patients with CD. The accumulation of sphingolipid compounds promotes inflammation.^54^ The results of our study indicated that the levels of the metabolite [SM(d18:0/16:1(9Z))] related to the sphingolipid signaling pathway and sphingolipid metabolism were decreased after 8 rounds of MSC treatment. Topological analysis of the pathway and KEGG rectangular tree diagram construction showed that linoleic acid metabolism was the main pathway showing changes between baseline and 8 MSC treatments. As an omega-6 fatty acid, linoleic acid is metabolized to arachidonic acid, a component of colonocyte membranes that assists in linoleic acid metabolism. A European prospective cohort study showed that a high dietary intake of linoleic acid more than doubled the risk of developing incident ulcerative colitis.^55^ The results of our study indicated that the levels of linoleic acid were reduced after 8 rounds of MSC treatment. MSC treatment inhibited pathways of linoleic acid metabolism. Moreover, correlations were observed between the fecal microbiota constituents and metabolites. Our results showed a possible link between the altered *Cetobacterium* abundance and linoleic acid metabolite levels in patients with CD who received 8 rounds of MSCs.

Whether the observed changes in microbiota and metabolites were secondary to the MSC infusions or a consequence of the improvement in inflammation is unclear. In equine keratinocytes, MSCs secrete CCL2, which stimulates the expression of antimicrobial peptides, and thus shows an increase in antimicrobial activity. The antimicrobial properties of MSC secretases involve both directly killing bacteria and indirectly stimulating the immune response of skin cells surrounding the bacteria.^56^ Therefore, infusions of MSCs may directly change the gut microbiota. In contrast, changes in the microbiota and metabolites may be a consequence of the improvement in inflammation. Due to a dysfunctional mucosal barrier, bacteria can invade the gut mucosa and thereby trigger excessive immune responses that lead to inflammation of the intestines.^57,58^ MSCs can promote the expression of the tight junction proteins claudin-1 and ZO-1 and thus improve the intestinal barrier function, which endows cell therapies based on MSCs with a unique ability to support the intestinal barrier of patients with IBD.^59^ Intestinal barrier recovery may induce changes in the microbiota and metabolites.

A limitation of this study was that the sample size was small for a phase I study. Furthermore, a controlled before-and-after study was performed without a control group. Finally, a complete analysis of the microbiome must also examine the virome and fungome in addition to the bacterial genome. The greatest strength of the study may be its innovativeness. Our study is the first to document changes in the gut microbiota of patients with CD after MSC treatment. We applied 16S rRNA amplicon profiling and nontargeted metabolomics. We observed negative correlations between the *Cetobacterium* abundance and the CDAI score and linoleic acid levels. Additionally, the levels of linoleic acid were reduced after 8 rounds of MSC treatment.

## Conclusion

In summary, this was a small, short-term, observational study of patients with CD. MSC infusions in patients with relapsing refractory CD were safe and well tolerated. A possible link between the altered *Cetobacterium* abundance and linoleic acid metabolite levels was observed in patients with CD who received MSCs. This study enabled an understanding of both the gut microbiota response and bacterial metabolites to obtain more information about host-gut microbiota metabolic interactions in the short-term response to MSC treatment.

## Supplementary Material

szad036_suppl_Supplementary_Table_S1

szad036_suppl_Supplementary_Table_S2

## Data Availability

The datasets used and/or analyzed during the current study are available from the corresponding author upon reasonable request. The raw data generated by 16S rRNA gene sequencing have been uploaded to the NCBI BioProject database (Submission ID: SUB12171737, Accession number: PRJNA892439). Raw LC-MS data have been uploaded to the online MetaboLights repository (ID: MTBLS6288). The data will be released to the public when the paper is published.
