# Supplementary material for: Short-Term Clinical Response and Changes in the Fecal Microbiota and Metabolite Levels in Patients with Crohn’s Disease After Stem Cell Infusions
Source: Stem Cells Transl Med. 2023 Jul 3;12(8):497–509. doi: 10.1093/stcltm/szad036 (PMC10427961; doi:10.1093/stcltm/szad036)
Supplement: szad036_suppl_Supplementary_Table_S1 [file szad036_suppl_supplementary_table_s1.docx]

**Supplementary Table1** Clinical parameters before and after MSC infusions

| **Number of patients enrolled** | **Before MSC infusion (baseline) (n=8)** | **After 4 MSC infusions**  **(n=8)** | **After 8 MSC infusions (n=7)** | **Statistics** |
| --- | --- | --- | --- | --- |
| **Weight (kg)** | 51.0±7.8 | 52.4±8.0 | 55.0±9.1** | RMANOVA  F=19.372  P<0.001 |
| **BMI (kg/m^2^)** | 20.0±2.6 | 20.5±2.7 | 21.5±3.0 | RMANOVA  F=0.371  P=0.697 |
| **ESR (mm/h)** | 19.1±15.0 | 17.6±15.5 | 14.5±11.1* | RMANOVA  F=4.571  P=0.033 |
| **CRP level (mg/L)** | 12.2±10.7 | 8.1±9.1* | 3.1±2.0* | RMANOVA  F=5.424  P=0.018 |
| **CDAI score** | 228.9±29.5 | 193.1±30.2* | 150.1±36.7*** | RMANOVA  F=28.694  P<0.001 |

*The results are presented as the means±SDs. Repeated measures analysis of variance (RMANOVA) was used to compare data obtained after 4 and 8 MSC infusions with the baseline data. The Bonferroni test was used for multiple comparisons: *P<0.05, **P<0.01, and ***P<0.001. Body mass index (BMI), erythrocyte sedimentation rate (ESR), C-reactive protein (CRP), and Crohn’s disease activity index (CDAI).
