# Supplementary material for: Short-Term Clinical Response and Changes in the Fecal Microbiota and Metabolite Levels in Patients with Crohn’s Disease After Stem Cell Infusions
Source: Stem Cells Transl Med. 2023 Jul 3;12(8):497–509. doi: 10.1093/stcltm/szad036 (PMC10427961; doi:10.1093/stcltm/szad036)
Supplement: szad036_suppl_Supplementary_Table_S2 [file szad036_suppl_supplementary_table_s2.docx]

Supplementary **table 2. Clinical features of patients experiencing relapse**

| **Patient number** | **Time to relapse (months after the last MSCs infusion)** | **Clinical manifestations of relapse** | **Therapeutic options following relapse** |
| --- | --- | --- | --- |
| #1 | 24 | Recurrence of anal fistula | Infliximab |
| #2 | 21 | haematochezia | Infliximab |
| #4 | No relapse | - | **-** |
| #5 | 3 | Perianal abscess | Infliximab |
| #6 | 27 | Loss in weight | Vedolizumab |
| #7 | 3 | Perianal abscess | Vedolizumab twice, but replaced, followed by ustekinumab |
| #8 | 23 | Intestinal obstruction | Colectomy |

* Luminal Crohn’s disease relapse is assessed by lower endoscopy.
